# Uterine Artery Doppler Ultrasonography for First Trimester Prediction of Preeclampsia in Individuals at Risk from Low-Resource Settings

**DOI:** 10.3390/medicina56090428

**Published:** 2020-08-26

**Authors:** Mihaela Oancea, Mihaela Grigore, Razvan Ciortea, Doru Diculescu, Diana Bodean, Carmen Bucuri, Stefan Strilciuc, Maria Rada, Dan Mihu

**Affiliations:** 1 Department of Obstetrics and Gynecology, “Iuliu Hatieganu” University of Medicine and Pharmacy, 400012 Cluj-Napoca, Romania; mihaelaoancea321@yahoo.com (M.O.); r_ciortea@yahoo.com (R.C.); ddiculescu@yahoo.com (D.D.); dianabodean13@gmail.com (D.B.); cbucurie@yahoo.com (C.B.); mpr1388@yahoo.com (M.R.); dan.mihu@yahoo.com (D.M.); 2 Department of Obstetrics and Gynecology, “Grigore T Popa” University of Medicine and Pharmacy, 700115 Iasi, Romania; 3 Department of Neurosciences, “Iuliu Hatieganu” University of Medicine and Pharmacy, 400012 Cluj-Napoca, Romania; stefan.strilciuc@ssnn.ro; 4 RoNeuro Institute for Neurological Research and Diagnostic, 400354 Cluj-Napoca, Romania

**Keywords:** preeclampsia, doppler ultrasonography, pulsatility index, pregnancy

## Abstract

*Background and objectives:* The objective of this study was to evaluate the potential of first trimester uterine artery Doppler ultrasonography for the early prediction of preeclampsia (PE), in at-risk pregnant women. *Materials and Methods:* This was a prospective longitudinal study, including 120 Caucasian pregnant women with risk factors for PE. The potential of pulsatility indexes (PI) and notch was assessed as a tool for preeclampsia screening. *Results:* Doppler examination of the uterine artery performed early at 11–14 WA allows the detection of pregnancies that will develop PE with a sensitivity of 61.5% and a specificity of 63.8% based on PI analysis. Predictive power increases slightly by adding bilateral notch (sensitivity = 65.4%; specificity = 66%). *Conclusions:* Uterine artery Doppler examination is an effective non-invasive screening test for the development of PE in pregnancies at risk, particularly appropriate in health systems with limited means of evaluating other biomarkers.

## 1. Introduction

Preeclampsia (PE) is one of the most severe complications of pregnancy and remains a principal cause of maternal–fetal mortality and morbidity worldwide. The prevalence of the disease is estimated at 3–5% of all pregnant women, representing the most frequent medical complication during pregnancy [1].

Recent years have seen important progress in understanding the pathogenesis of this disorder, as well as its prevention [2]. Additionally, a wide range of potential biomarkers for prediction of preeclampsia have been studied: maternal characteristics (African-American ethnicity, body mass index, pregestational diabetes mellitus, systolic blood pressure, educational level), pregnancy-associated plasma protein A (PAPP-A), A Disintegrin and Metalloprotease 12 (ADAM-12), placental growth factor (PlGF), soluble fms-like tyrosine kinase 1 (sFlt-1), placental protein 13 (PP13), proteomics studies, Doppler ultrasonography, and many others [3,4,5,6,7,8].

The pathophysiological events that lead to the development of PE occur in response to abnormal placentation. Despite an adaptive response in the early phase, systemic circulation alterations and an imbalance of vasoactive factors occur. Trophoblast invasion failure, supposedly due to the interaction between exposure to risk, presence of polymorphic genes and several other factors (vasoactive and vascular remodeling proteins, thrombophilia, hypofibrinolysis, oxidative stress, lipid metabolism, endothelial injury and immunogenetic factors), deters physiological remodeling in the spiral arteriolar walls [9]. Changes in these physiological processes determine high resistance circulation in the terminal vascular territory of spiral arteries. Moreover, terminal circulation maintains its reactivity to vasomotor agents [10,11].

These hemodynamic changes occur before the clinical onset of PE and may be assessed by Doppler examination. Quantitative and qualitative analysis of vascular flows by ultrasonography is reliable and reproducible, having been reported to detect 75% of cases of preterm (before 37 weeks of gestation) preeclampsia, as part of a multidimensional ensemble of screening indicators [12].

Nevertheless, the translation of rich biomarker evidence into clinical practice remains difficult in low-resource settings. Unfortunately, countries with low expenditure for health do not have the resources to implement changes to common practice that incur additional cost. Impoverished countries require novel approaches to adapt existing evidence in medicine in order to benefit the largest possible number of people [13].

The uterine artery has been extensively evaluated in PE, indicating maternal vascular status by assessment of pulsatility index (PI), resistivity index (RI), and the presence of early diastolic notch. The persistence of early diastolic notch or abnormal velocity aspects of the blood flow have been associated with trophoblast invasion failure [14,15,16,17].

In this context, we explored the hypothesis that a basic, routinely performed procedure—first-trimester ultrasound monitoring—may provide a window of opportunity for screening for preeclampsia using a standalone biomarker, in pregnant women with known risk factors for the disorder. Therefore, we studied the potential of first trimester uterine artery Doppler ultrasonography for the early prediction of PE, in at-risk pregnant women.

## 2. Materials and Methods

A prospective longitudinal study of Caucasian pregnant women with risk factors for PE was waived by the institutional review board of the Iuliu Hatieganu University of Medicine and Pharmacy in Cluj-Napoca. Study inclusion criteria required the presence of at least one of the following risk factors: primiparity; history of PE in previous pregnancies; family history of PE; chronic arterial hypertension; renal diseases; diabetes mellitus; systemic lupus erythematosus; antiphospholipid syndrome; thrombophilia; history of obstetric disorders (fetal hypotrophy, oligoamnios, perinatal mortality, premature separation of the normally implanted placenta); obesity (BMI > 30 kg/m^2^); maternal age (<18 years or >40 years). Exclusion criteria were infections, recent treatment with non-steroidal anti-inflammatory drugs and corticosteroids (14 days prior to inclusion), chronic inflammatory diseases, multiple pregnancies, and fetal abnormalities.

All patients included in the study signed an informed consent. A standardized work chart was drawn up, which recorded anthropometric data, family history, personal physiological and pathological history, obstetric parameters (gestation, parity, date of the last menstruation, first fetal movements, probable date of birth, evolution of previous pregnancies and births), ultrasound parameters in the first trimester (measurement of craniocaudal length (CCL), presence of embryonic cardiac activity, Doppler examination of the uterine artery).

For the diagnosis of PE, we used criteria proposed by the American College of Obstetricians and Gynecologists: blood pressure values of at least 140/90 mm Hg (2 examinations between 6 h and 7 days apart), proteinuria above 30 mg/dl (2 urine samples collected between 4 and 6 h apart). Moderate PE were defined as asymptomatic, presenting hypertension with values below 160/110 mm Hg and proteinuria [18]. Hypertension and proteinuria was observed after 20 weeks of pregnancy in previously (prior to pregnancy) normotensive women and normalized by 12 weeks postpartum. At the end of the study, the patients were divided into two groups depending on the pregnancy evolution. The basic parameter monitored in our study was the pulsatility index (PI = S-D/Mean) and the presence of bilateral notch in the uterine artery.

The results obtained were correlated with the subsequent evolution of PE or its complications: eclampsia, PSNIP, HELLP syndrome, perinatal fetal distress (fetal hypotrophy, antepartum or intrapartum fetal death, Apgar score at birth < 7).

### Doppler Ultrasound Procedures

Ultrasonography was performed using a General Electric Voluson 730 ultrasound machine, equipped with a convex abdominal probe of 2.0–5.0 MHz and an endovaginal probe (4–10 MHz). The procedure required an angle of less than 60° between the incident ultrasound beam and the studied vessel. Indicators were calculated on samples of minimum 5 waves with identical appearance and a clearly defined outline of the spectrum.

Uterine and fetal vessels were identified using color Doppler and power Doppler, then quantitative and qualitative evaluation was performed by pulsed Doppler. The velocimetric evaluation of the uterine artery reflects the hemodynamic particularities of the maternal side as part of the maternal–fetal–placental unit.

As part of routine ultrasound at 11–14 weeks, a sagittal section of the cervix was obtained. The probe was swept laterally until the paracervical plexus was identified. Color Doppler was used to identify the uterine artery at the cervico-corporeal junction. The measurements were performed at this point, before the branching of the uterine artery into the arcuate arteries. Pulsatility indices were measured bilaterally and the mean PI was calculated. The presence or the absence of bilateral early diastolic notch was also recorded. Abnormal uterine artery Doppler appearance was defined as the presence of bilateral notch and/or a mean PI > 95th percentile. For PI interpretation, we used the normative reference curves by age (first trimester 95th percentile value = 2.6).

A binary logit model was used to assess whether Doppler changes (PI > 95th percentile or bilateral notch) in the first and/or second trimester are correlated with the probability of development of PE. A *t* test was used for the study of the statistical significance of coefficients. A variable is considered to be statistically significant if the value of *p* < 0.05. The predictive power of the functions used was assessed by the ROC (Receiver Operating Characteristics) curve. Statistical processing was performed with the STATA 9.1 software (StataCorp, College Station, TX, USA).

## 3. Results

### 3.1. Study Population

The study enrolled 120 pregnant women who met inclusion and exclusion criteria. Group I was comprised of 26 patients who developed PE (21.6%), out of which nine had severe and 17 had moderate forms. Of the nine pregnant women with the severe form of PE, four also had intrauterine growth restriction (IUGR). Group II was comprised of 94 pregnant women who did not develop PE, following a physiological evolution of the pregnancy. Basic demographic characteristics of patients are available in Table 1.

We analyzed the predictive power of Doppler changes in the uterine artery in the first trimester of pregnancy (11–14 WA). The difference between the mean PI value between groups was statistically significant (*p* = 0.012). Of all pregnant women included in the study, on Doppler examination of the uterine artery performed in the first trimester of pregnancy, 16.6% (20/120) had a pathological PI (>2.6). Of these, 26.9% (7/26) belonged to group I, subsequently developing PE, and 13.8% (13/94) were assigned to group II, without developing this disease. The increased PI values in the first trimester are statistically significant (Table 2).

### 3.2. PI Alone as a First-Trimester Biomarker for Preeclampsia

For the cut-point c = 0.22 in which sensitivity is closest to specificity, prediction was correct for 16 of 26 patients, sensitivity being 61.5% among patients with PE. Among patients without PE, prediction was correct for 60 of 94 patients, specificity being 63.8%. On ultrasound examination in the first trimester of pregnancy, 45.8% (55/120) of patients in the studied group had bilateral notch. Of these, 61.5% (16/26) developed PE during pregnancy evolution, being included in group I, and 41.5% (39/94) did not develop this disease and were included in group II. An analysis of the global changes in the Doppler parameters studied in the first trimester (pathological PI and notch) shows that of the 120 pregnant women included in the study, 63 (52.5%) had altered Doppler. Of these, 17 patients belonged to group I, representing 65.4% of it.

**Figure 1:**
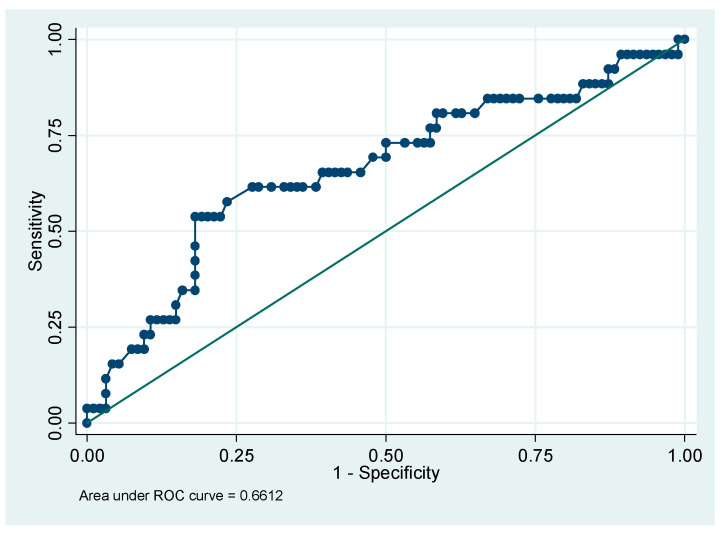
Receiver operating characteristic (ROC) curve associated with the binary logit model. Dependent variable: PI in the first trimester. PI: pulsatility index.

The predictive power of PI values in the first trimester is moderate (Figure 1—AUROC = 0.6612). In conjunction with result of the linear logistic regression (Table 3), preeclampsia screening based only on PI values in the first trimester is not advised.

### 3.3. PI and Notch as First Trimester Biomarkers for Preeclampsia

For the cut-point c = 0.25 in which sensitivity is closest to specificity, among patients with PE, prediction was correct for 17/26 patients (sensitivity = 65.4%). Among patients without PE, prediction was correct for 62/94, specificity being 66.0%. For all patients, the percentage of correct predictions was 65.8%.

**Figure 2:**
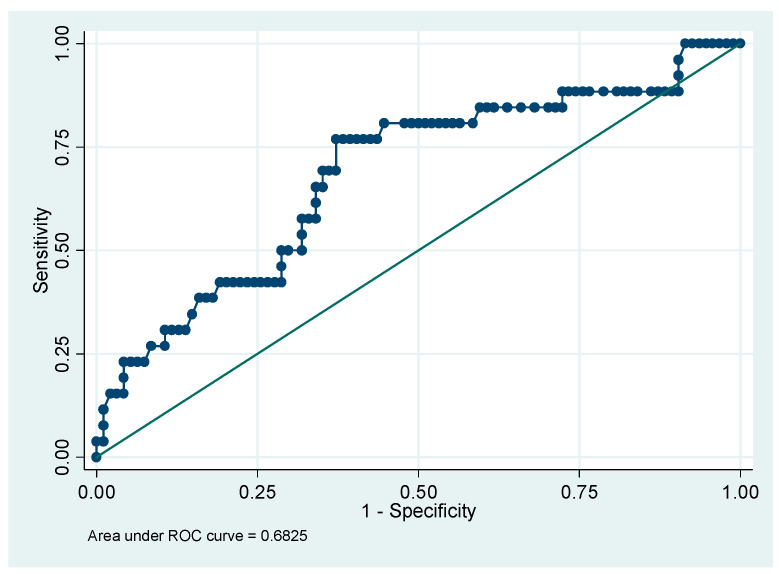
ROC curve associated with the binary logit model. Explicative variables: PI and notch in the first trimester.

The predictive power of PI values and the presence of notch in the first trimester is slightly higher (Figure 2—area under the ROC curve (AUROC) = 0.6825) than when prediction is based on PI alone in the first trimester. In conjunction with the result of regression (Table 4), it shows that a suspicion of PE based only on PI values and the frequency of notch in the first trimester is risky.

## 4. Discussion

Of the 120 pregnant women included in the study, 26 (21.6%) developed PE during pregnancy. It should be noted that the presence of vascular pathology prior to pregnancy or obstetric accidents unexplained at the time of their occurrence increase the risk of PE. The results obtained demonstrated the presence of significant hemodynamic changes in uterine circulation in the first half of pregnancy, in accordance with other existing studies [19,20,21,22]. The trophoblast invades the decidual part of spiral arteries between 8 and 12 WA [23]. Changes in protein and steroid hormones can also play an important role in these vascular alterations [24]. In addition, intervillous circulation stabilization and marked changes in umbilical circulation occur during this early stage of pregnancy [25]. Our study allowed describing the differences in the sequence of changes in uterine artery Doppler waves in normal pregnancy and pregnancy complicated by hypertensive pathology. We monitored both the mean uterine artery PI and the presence of bilateral notch, which allowed us to analyze them separately. Early diastolic notch is characteristic of high resistance vessels and quantifies the vessel elasticity. It seems to be dependent on maternal–placental interactions, more probably reflecting the decidualization of spiral arteries [26].

On the other hand, uterine artery PI reflects total measurable distal vascular resistance and provides information about placental volume and the cross-section of placental vessels [27]. Recent studies have evidenced that predisposing maternal factors may induce functional atherosclerotic and vasoactive changes in spiral arteries, which are more or less transformed into uteroplacental arteries, resulting in preeclamptic syndrome [28,29,30].

Our study demonstrated that patients with high uterine artery PI and bilateral notch in the first trimester of pregnancy had the highest risk of developing PE. The data obtained in this study show the fact that in PE screening, in patients at high risk, Doppler examination of the uterine artery performed early at 11–14 WA allows the detection of pregnancies that will develop PE with a sensitivity of 61.5% and a specificity of 63.8% based on PI analysis. If the presence of bilateral notch is added to this parameter, the predictive power increases slightly (sensitivity = 65.4%; specificity = 66%). The results of our study are in agreement with other studies which, analyzing Doppler changes in the first trimester of pregnancy, reported similar detection rates of overall PE, with a higher prediction for early onset PE (<34 WA) [31,32,33,34,35,36,37].

One of the limitations of our study was the small number of patients. This is why only the prediction of overall PE was considered. Early detection of cases with a risk of developing PE will allow the application of prophylactic treatment. The cases detected in this way are those that will develop severe complications and could be further subjected to an intensive monitoring program.

Currently, the ability of individual markers to predict PE remains modest. The use of first trimester bioumoral parameters and uterine artery Doppler indices has the highest potential to become a screening method in low-resource settings. Adequate prospective studies using standardized methods are necessary in the future to evaluate the choice of parameters and strategies for an association aimed at obtaining the best predictive methods. Improving the knowledge of the pathogenesis of complications during pregnancy will facilitate the development of new prediction and prevention methods.

## 5. Conclusions

Uterine artery Doppler examination is an effective non-invasive screening test for the development of PE in pregnancies at risk, particularly appropriate in health systems with limited means of evaluating other biomarkers. Doppler evaluation in pregnancies with PE allows adequate monitoring, with a judicious choice of the time and way of delivery, a decrease in the number of emergency cesarean sections for fetal distress, and an improvement of perinatal outcomes.

## Figures and Tables

**Table 1:** Maternal and newborn characteristics of the study population.

Indicator	N	% Total
**Blood Pressure**		
Normal	94	78.33%
≥160/110 mmHg	9	7.50%
SBP 140–160 mmHg and DBP 90–110 mmHg	17	14.17%
**Body Mass Index**		
≥30	20	16.67%
<30	100	83.33%
Age (years)		
18–29	51	42.50%
30–40	60	50.00%
over 40	9	7.50%
**Gestational Age at Birth (Weeks)**		
<34 weeks	5	4.17%
34–37 weeks	25	20.83%
over 37 weeks	90	75.00%
**Birth Weight (Grams)**		
0–1500	4	3.33%
1501–2000	11	9.17%
2001–2500	32	26.67%
2501–3000	41	34.17%
3001–3500	28	23.33%
3501–4000	3	2.50%
4001+	1	0.83%
**Apgar Score**		
3	4	3.33%
4	4	3.33%
5	8	6.67%
6	13	10.83%
7	24	20.00%
8	17	14.17%
9	16	13.33%
10	34	28.33%

**Table 2:** Descriptive statistics for pulsatility index in the first trimester.

	Group I (Preeclampsia)	Group II (Control)
Mean	2.25	1.97
Standard deviation	0.54	0.49
Minimum	0.92	0.87
Q1	1.87	1.69
Median	2.40	1.93
Q3	2.62	2.23
Maximum	3.30	3.04
*t* test (*p* value)	0.012	

**Table 3:** Results of binary logit regression, PI in the first trimester.

	Coefficient	Std. Error	Z *	*p* Value
PI first trimester	1.129	0.467	2.42	0.016
Const.	−3.664	1.044	−3.51	0.000

* Regression coefficient divided by standard error. PI: pulsatility index.

**Table 4:** Results of binary logit regression, with the explicative variables PI and notch in the first trimester.

	Coefficient	Std. Error	Z	*p* Value
PI first trimester	1.056	0.472	2.24	0.025
Notch first trimester	0.708	0.466	1.52	0.128
Const.	−3.875	1.068	−3.63	0.000
